# Effect of Using a Cardiac Catheterization Table-Stabilizing Stick on the Quality of Cardiopulmonary Resuscitation in the Cardiac Catheterization Laboratory: A Simulation-Based Study

**DOI:** 10.1155/2019/6303978

**Published:** 2019-08-01

**Authors:** Tsuyoshi Yamada, Morihiro Ito, Hisako Urai, Yumiko Ueda, Hiroaki Maki, Reizo Baba

**Affiliations:** ^1^Graduate School of Life and Health Sciences, Chubu University, Aichi, Japan; ^2^Department of Biomedical Sciences, College of Life and Health Science, Chubu University, Aichi, Japan; ^3^Department of Lifelong Sports and Health Sciences, College of Life and Health Science, Chubu University, Aichi, Japan; ^4^Faculty of Nursing, Gifu Shotoku Gakuen University, Gifu, Japan; ^5^Department of Radiological Technology, Mie University Hospital, Mie, Japan

## Abstract

Rapid defibrillation and high-quality cardiopulmonary resuscitation (CPR) are necessary for patients with cardiopulmonary arrest, one of the most serious and frequently encountered complications in cardiac catheterization laboratories. However, when the catheterization table is withdrawn from its neutral position for fluoroscopy, it is unstable and unsuitable for resuscitation because of its cantilever structure. To stabilize the table in its withdrawn position, the use of a table-stabilizing stick might improve CPR quality. To investigate the effect of using a cardiac catheterization table-stabilizing stick on CPR quality, a CPR simulation mannequin was placed on a cardiac catheterization table that was withdrawn from the C-arm of the X-ray machine. CPR quality was assessed with or without the use of a table-stabilizing stick under the table. The CPR quality assessment (Q-CPR) scores were 79.6 ± 11.4% using the table-stabilizing stick and 47.7 ± 30.3% without the use of the stick device (p = 0.02). In this simulation-based study, the use of a table-stabilizing stick in a cardiac catheterization table withdrawn from the C-arm of the X-ray machine improved the quality of CPR.

## 1. Background

Catheter interventions have a long history. In 1964, Dotter et al. succeeded in treating occlusive lesions in arteries of the lower extremities [1]. Later, Judkins developed a coronarography procedure that was made available to the public in 1967 [2], and Gruentzig performed percutaneous transluminal coronary angioplasty involving the insertion of a balloon catheter and announced its outcome in 1977 [3].

Since then, catheter interventions have spread rapidly worldwide, which led to the development of a procedure to dilate the coronary arteries, a better understanding of the pathological conditions of coronary artery atheroma, and improvement of long-term prognosis by actively adapting these interventions for acute myocardial infarction. Progress in catheter technology developed for the coronary arteries, along with better understanding of pathological conditions, has improved vascular treatment (such as in carotid arteries, lower extremity arteries, and renal arteries), promoted alternative options instead of surgical treatment for valvular heart disease, improved the nondrug treatment of cardiac arrhythmias, and allowed a catheter to reach all parts of the human body—in other words, the era of using catheter interventions in different treatments has arrived. Currently, catheter interventions, which have been remarkably developed by improvement in device quality and accumulation of experiences, are being performed in 200,000 patients with ischemic heart disease per year in Japan.

However, every medical practice, regardless of the degree of invasiveness, is associated with the risk of complications. Cardiopulmonary arrest is one of the most serious complications during cardiac catheterization. In the 1960s, the mortality rate for diagnostic catheterization was found to be 1.0% [4]. In a study on diagnostic catheterization performed between 1979 and 1981, which was first registered in the Society for Cardiovascular Angiography and Interventions (SCAI), the procedure-related mortality rate of 53,581 patients was found to be 0.14% [5]. However, a similar study of 222,553 patients who underwent catheterization between 1984 and 1987, which was also presented at the SCAI, showed that the mortality rate associated with diagnostic catheterization was reduced by up to 0.1% [6]; the mortality rate was further reduced by 0.08% according to data obtained from the SCAI in 1990 [7]. According to Singh et al. of Mayo Clinic, who presented 25-year data (1979-2004) on >24,000 patients treated with percutaneous coronary intervention (PCI), the complexity of comorbidities and lesions increased with age during the 25-year period [8]. Brennan et al. reported on PCI-related mortality rate among 1,208,137 patients who underwent PCI between 2009 and 2011, and their data, which reflected disease severity, showed an overall hospital mortality rate of 1.4% [9].

According to Addala et al., who reported outcomes of a single-center study on the PCI database of >19,000 patients [10], although cardiopulmonary arrest or ventricular fibrillation (VF) occurred in 164 patients (0.84%), the patients were defibrillated within a minute; therefore, all 164 patients were resuscitated. In another study of 3,065 patients with myocardial infarction who underwent primary angioplasty, Mehta et al. also reported on incidence rate of ventricular tachycardia/VF in patients treated with PCI [11] and pointed out that cardiac arrest occurred in 4.3% of patients. Although the incidence rates in these findings are not high, it can be easily assumed that some of the patients had cardiopulmonary arrest, which is a severe complication during cardiac catheterization.

The American Heart Association guidelines for cardiopulmonary resuscitation (CPR) recommend that, in order to ensure good results, rapid defibrillation and high-quality CPR should be provided to patients in cardiopulmonary arrest. Rapid defibrillation and high-quality CPR are mandatory for patients with cardiac catheters who are in cardiac arrest [12]. However, in the cardiac catheterization laboratory, CPR procedures are usually performed on an unstable catheterization table, which often impairs the quality of chest compression [13]. Most CPR procedures on a catheterization table are performed when the patient's condition suddenly changes during the catheterization procedure. In addition, since cardiac catheterization is intended to treat cardiovascular conditions including those causing cardiac arrest, it is necessary to continue the CPR procedure in case of cardiac arrest without interrupting the catheter technique. Hence, the implementation of CPR can be difficult when the patient's heart is undergoing fluoroscopy during cardiac catheterization.

Under these circumstances, we have developed a simple stick device to stabilize the catheterization table and improve the quality of chest compression. This study thus aimed to investigate the effect of using the table-stabilizing stick on the quality of CPR performed on a simulation mannequin.

## 2. Materials and Methods

This study was approved by the Ethics Committee of Chubu University (approval no. 260035-2). Informed consent was obtained from all participants who performed the procedures.

### 2.1. Research Participants

Twelve healthy university students (average height: 171.6 ± 4.3 cm) participated in this study to perform the CPR procedures. All participants were students who expressed interest in emergency life-saving measures and were actively involved in simulation-based training and research on CPR aimed for emergency life-saving. All participants who performed procedures provided informed consent to be involved in the study.

### 2.2. Measurements of CPR Quality

**Figure 1:**
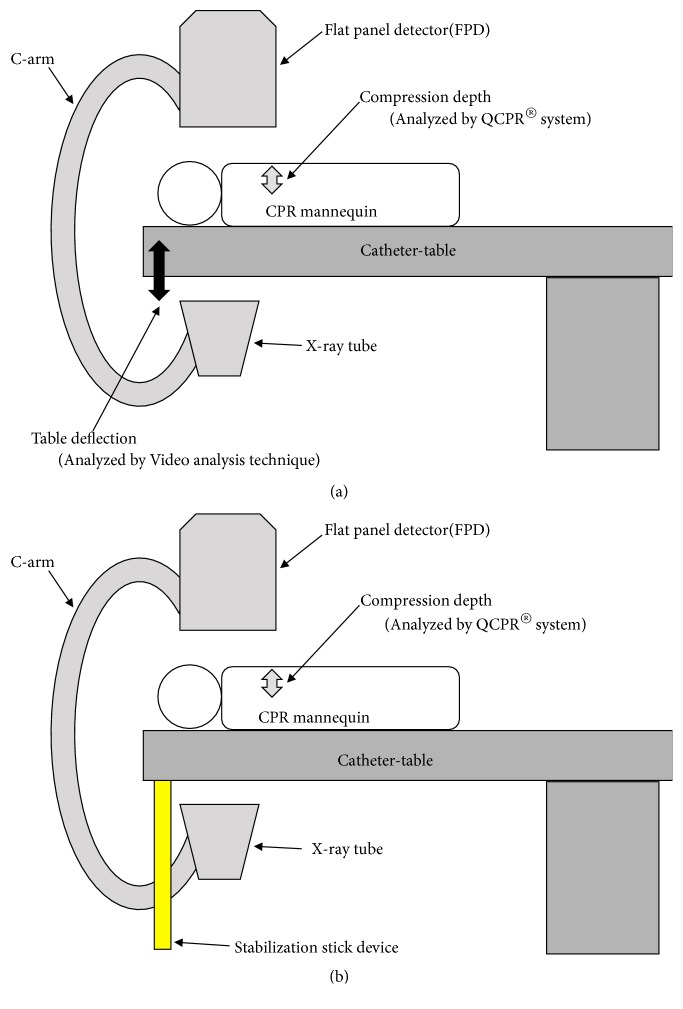
Diagrams of the study setting. (a) When the table-stabilizing stick is not installed under the cardiac catheterization table. (b) When the table-stabilizing stick is installed under the cardiac catheterization table. Compression depth was measured by the QCPR® System, and table deflection was measured by video analysis.

Figure 1 shows the diagrams of the study setting. CPR quality was measured using the QCPR® System, which consisted of a simulation mannequin (Resusci Anne®, Laerdal Medical, Norway) and real-time training software (QCPR®). The system can measure and assess hand positions and compression rate and depth, being able to distinguish between fully released, shallow, and sufficiently deep compressions, and can provide a comprehensive qualitative assessment of CPR in the form of a compression score (also called Q-CPR) (in %) [14]. Because this study focused on chest compressions, parameters related to chest compressions were studied using a mode to assess chest compressions only. The parameters were set according to the Japan Resuscitation Council. The participants only performed CPR. The duration of each CPR procedure was 2 minutes.

### 2.3. Video Analysis

A video analysis technique was used for assessing the deflection of the catheterization table during chest compression. The upper edge of the catheterization table was marked with vinyl tape, and during chest compression, all images including those of the marked edge were taken with a Hi-Vision Memory Movie Camcorder (shutter speed: 1/4000, model GZ-V590; JVC, Japan) under the same conditions (i.e., same camera position, object location, etc.). A two-dimensional video analysis with Dartfish® software was used to determine the deflection (in millimeters) of the angiography table with or without the use of the table-stabilizing stick. To determine deflection, we recorded movement of the table, from the flat state to the state of maximum chest compression. We measured the travel distance of the table from the recording with software.

### 2.4. Catheterization Laboratory Environment

We used the X-ray system (Philips Allura Xper FD10/10 biplane), which is a cardiac angiography system.

### 2.5. Table-Stabilizing Device

**Figure 2:**
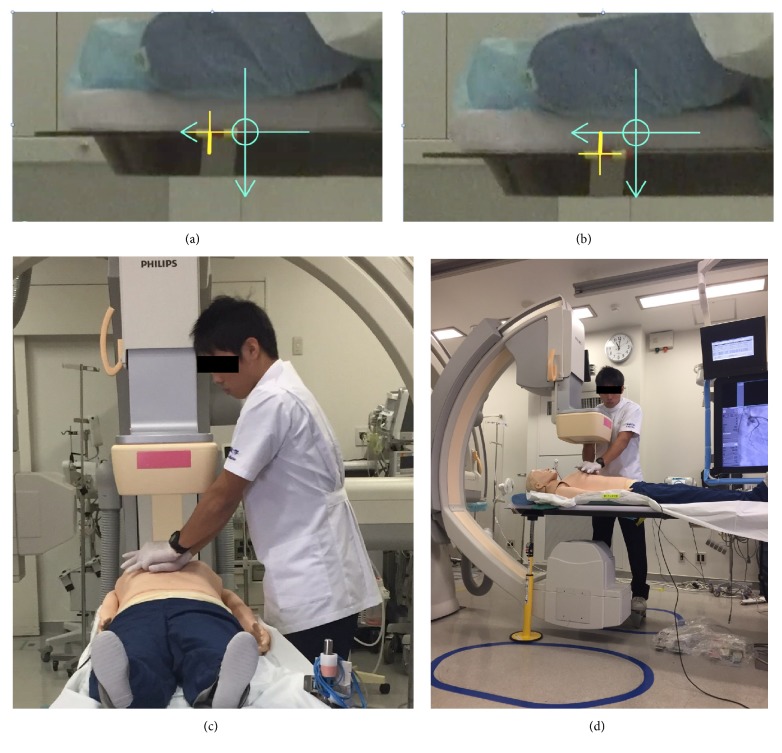
Alignment of the video camera during CPR simulation and photographs of the procedure. (a) Yellow crosshair mark indicates the height of the angiography table at the time of a fully released chest compression. (b) Distance between the light blue crosshairs and yellow crosshairs indicates deflection of the catheterization table during a chest compression. (c) Position of the arms when the participant performs CPR on the catheterization table. (d) A CPR procedure performed with the use of a table-stabilizing stick under the catheterization table. The chest of the mannequin is located between the X-ray tube and the flat panel detector. Therefore, it is impossible to push the chest vertically.

We used a table-stabilizing stick (ANG-MU11; Mie Metal Industry Co., Ltd., Japan) to prevent deflection of the catheterization table; see Figure 2(d)).

### 2.6. Statistical Analysis

Data were analyzed by applying the Student's t-test on repeated measurements. Measurements under two conditions, (a) with and (b) without the use of the table-stabilizing stick installed under the catheterization table, were compared. Results were expressed as the means ± standard deviations (SDs). All statistical analyses were performed with SPSS software version 24.

## 3. Results

### 3.1. Analysis of CPR Quality

When the table-stabilizing stick was installed, the QCPR® System showed a compression score of 79.6 ± 11.4%, which demonstrated that the CPR quality performed by the research participants was fairly good. Under the same conditions, the compression depth assessed using video analysis was 47.3 ± 2.9 mm. When the table-stabilizing stick was not installed, the QCPR® System showed a compression score of 47.7 ± 30.3%, which demonstrated poor CPR quality. Here, the compression depth assessed using video analysis was 40.8 ± 7.2 mm. Measurement of compression depth revealed a significant difference between CPR simulations performed with and without the use of the table-stabilizing stick (6.5 ± 7.7mm; P < 0.01; Table 1).

### 3.2. Video Analysis

When the table-stabilizing stick was not installed, the catheterization table showed a travel distance of 6.6 ± 1.9 mm, based on the two-dimensional motion analysis software Dartfish®. Meanwhile, when the table-stabilizing stick was installed, the catheterization table showed 0 mm of travel distance. A two-tailed Student's t-test showed a significant difference in the measurements of deflection (P < 0.01; Table 1).

## 4. Discussion

In this study, we have investigated the effect of installing a table-stabilizing stick on the quality of CPR, using the QCPR® System, which consisted of a mannequin and real-time training software. The use of the simple catheterization table-stabilizing stick has significantly improved the Q-CPR score that reflects CPR quality during cardiac catheterization. This research is simple and the results can be easily predicted. However, we feel that it is meaningful to prove this using a mannequin.

Cardiopulmonary arrest is one of the most serious and frequently encountered complications during cardiac catheterization [10, 11]. The mortality rate due to cardiopulmonary arrest during diagnostic cardiac catheterization is reported to be around 0.01% [5–7]. In another study, catheter interventions have been associated with higher mortality [9]. Thus, rapid defibrillation and effective resuscitation are mandatory for patients with cardiopulmonary arrest. However, during cardiac catheterization, the performance of CPR can be difficult due to the unstable cantilever position of the catheterization table and the need to withdraw the table from the C-arm of the X-ray machine. Our study has revealed that an average deflection of 6.6 cm of the catheterization table could reduce CPR quality and that such deflection could be prevented with the use of a table-stabilizing stick.

During cardiac catheterization, the catheterization table is designed to be withdrawn from the C-arm of the X-ray machine. Withdrawing the table can cause imbalance in load distribution around the table because of its cantilever design, wherein the load is supported by one fulcrum away from the center of gravity. During CPR, force from the hands of the practitioner performing the procedure is applied to the distal end of the table, which increases the load on that side and causes it to deflect from a normal flat angle, thus greatly reducing the quality of the CPR. Inserting a stick device to the distal end of the table when it is withdrawn from the C-arm provides an additional fulcrum for support, can eliminate deflection of the table during CPR (see Figure 1(b)), and thus can improve CPR quality.

Although the table-stabilizing stick used in this study prevented deflection of the angiography table and significantly improved CPR quality, the CPR quality (expressed by compression score) remained at 79.6 ± 11.4% and has not yet reached 100%. Such increase in SD to 11.4% could be attributed to the position of the flat panel detector and X-ray tube, which are located just above and below the mannequin's chest, respectively. Such structures make it difficult for the practitioner to push the chest vertically during chest compression, causing tilting of force applied to the chest (see Figures 2(c) and 2(d)). A study in piglets by Zuercher et al. showed that tilting during chest compression decreased cardiac output and impaired left ventricular myocardial blood flow during cardiac arrest [15].

Another possible method of performing effective chest compressions in the catheterization room is the use of mechanical chest compressors (e.g., LUCAS device) [16]. The device is useful for prolonged resuscitation efforts; however, initiating operation of the device takes at least several minutes. Moreover, the LUCAS device does not have X-ray transparency and thus prevents further catheterization procedures after cardiac arrest events. Meanwhile, the table-stabilizing stick device is quite simple and takes only a few seconds to install. Therefore, we recommend the use of this stick device in cardiac catheterization laboratories worldwide.

## 5. Limitations

Our study has several limitations. Firstly, it is a simulation-based study using only one type of mannequin. In a real clinical setting, complex factors exist, such as patient weight, size, body type, and chest compliance. However, it is impossible to conduct experiments in humans; hence, we believe the findings of this research remain significant. Secondly, only one type of catheterization table was used. As different catheterization machines with various catheterization tables exist, the use of one type of table could introduce some variability in the quality of compressions. Thirdly, we selected general university students to perform the CPR procedures, which may also introduce inconsistencies in CPR quality. However, to minimize such risk of variation, we only selected students who were routinely involved in and had adequate CPR training to become emergency life-saving practitioners. Such selection of students better represents the variance encountered in real clinical encounters and thus improves the generalizability of our findings. Fourthly, all research participants were men, which may possibly influence the generalizability of our findings. Finally, although the use of a mechanical chest compressor (e.g., LUCAS device) is useful for long-term resuscitation work, in this study the LUCAS device could not be used because it does not have X-ray transparency.

## 6. Conclusions

This study has shown that the use of a catheterization table-stabilizing stick improved the quality of CPR when the table was withdrawn from the C-arm of the X-ray machine. We therefore recommend the use of this stick device in cardiac catheterization laboratories.

## Figures and Tables

**Table 1:** Effect of the catheterization table-stabilizing stick on the quality of cardiopulmonary resuscitation (CPR).

	Table-stabilizing stick		Comparison
	Use	Nonuse	
Compression score (Q-CPR)^#^ (%)	79.6 ± 11.4	47.7 ± 30.3	31.9 ± 32.4
Depth (mm)	47.3 ± 2.9	40.8 ± 7.2	6.5 ± 7.7
Fully released chest compressions (%)	92.1 ± 8.3	96.3 ± 3.4	-4.1 ± 9.0
Sufficiently deep chest compressions (%)	36.4 ± 21.6	19.9 ± 17.3	16.6 ± 27.7
Table travel distance (mm)	0	6.6 ± 1.9	-6.6 ± 1.9

^#^P < 0.01 for use and nonuse of table-stabilizing stick.

## Data Availability

The data used to support the findings of this study are available from the corresponding author upon request.

## Abbreviations

CPR: Cardiopulmonary resuscitation.

## Additional Points

We carried out the study with respect for human rights.
